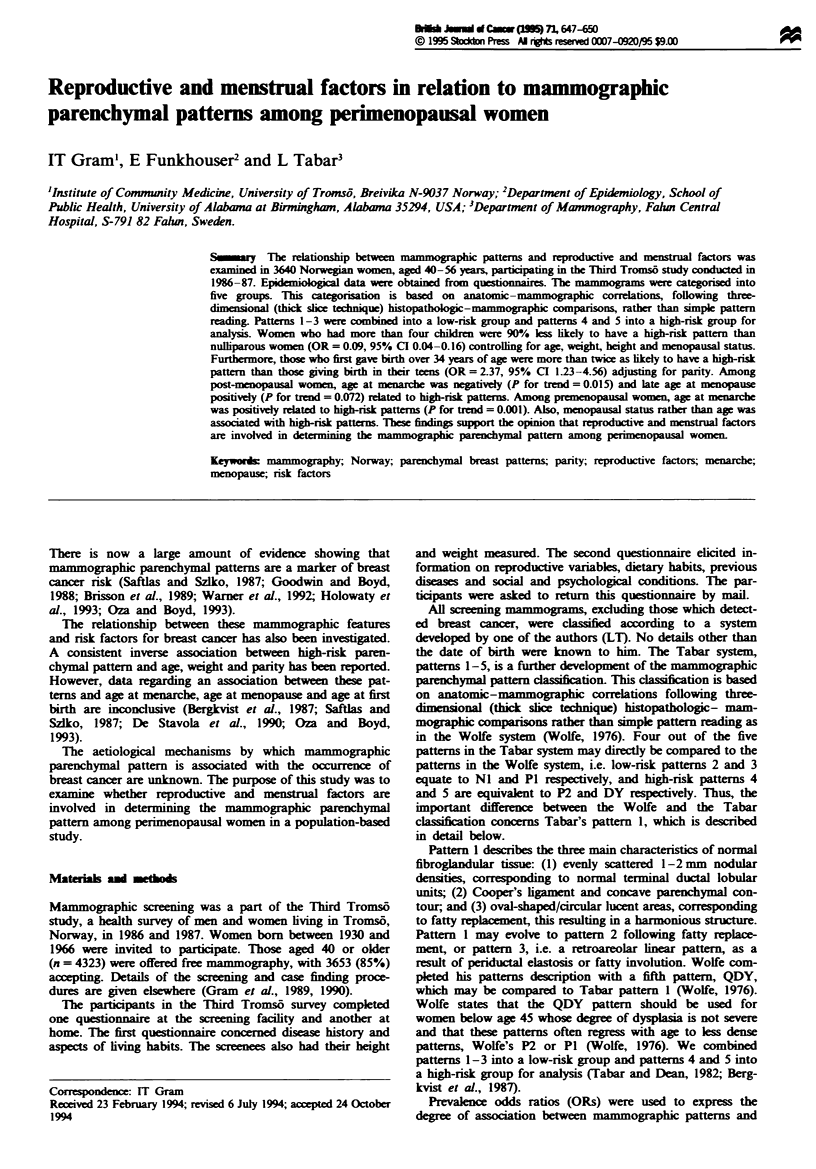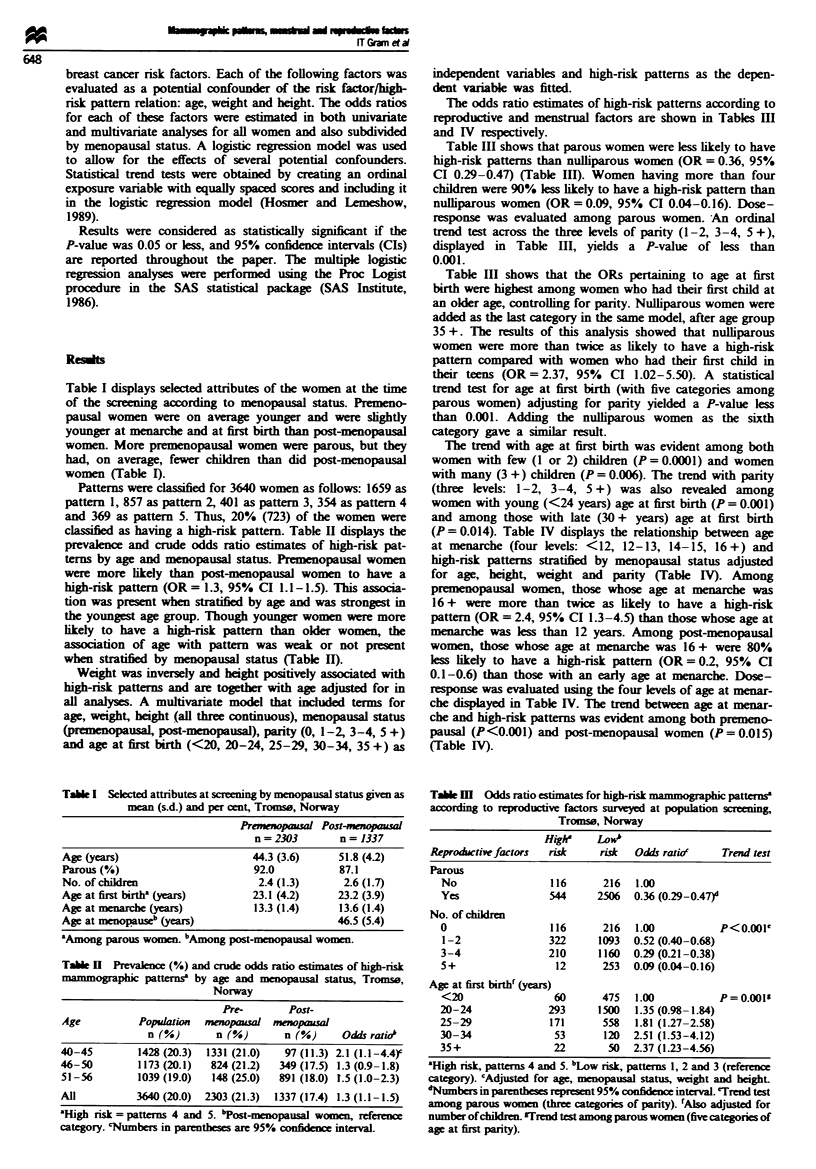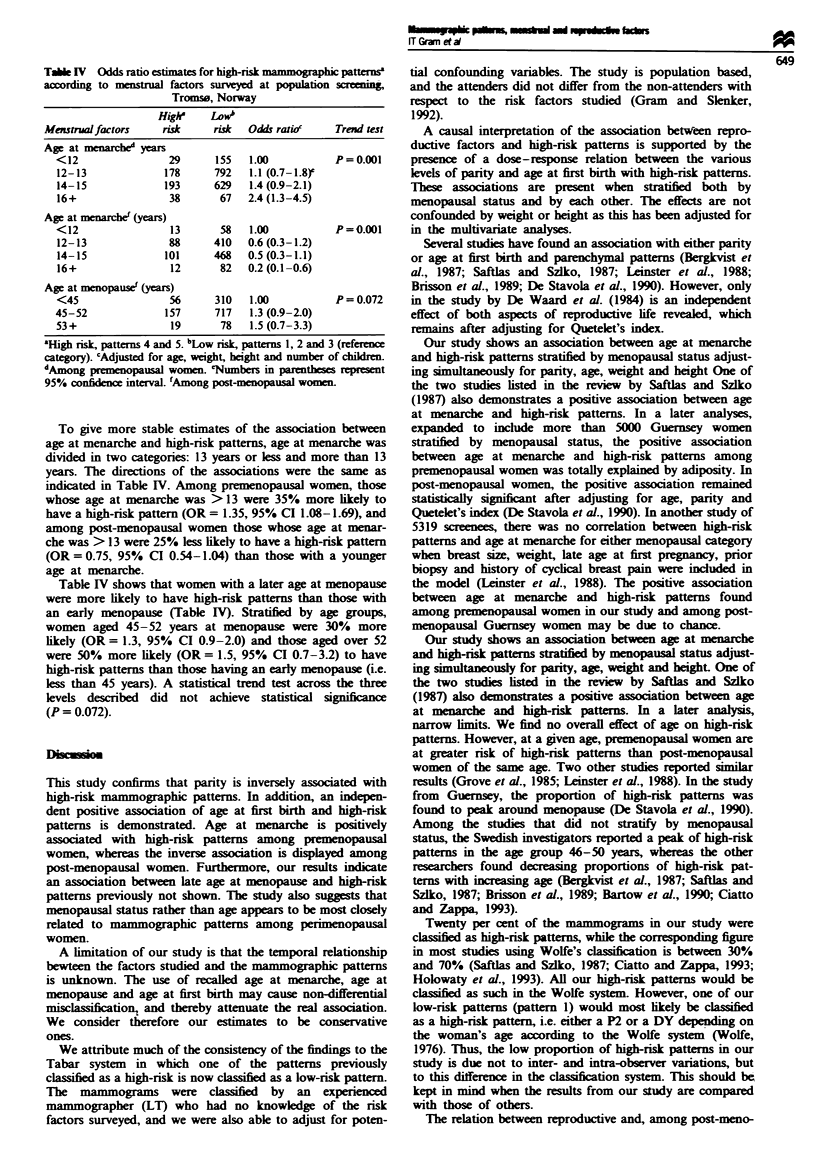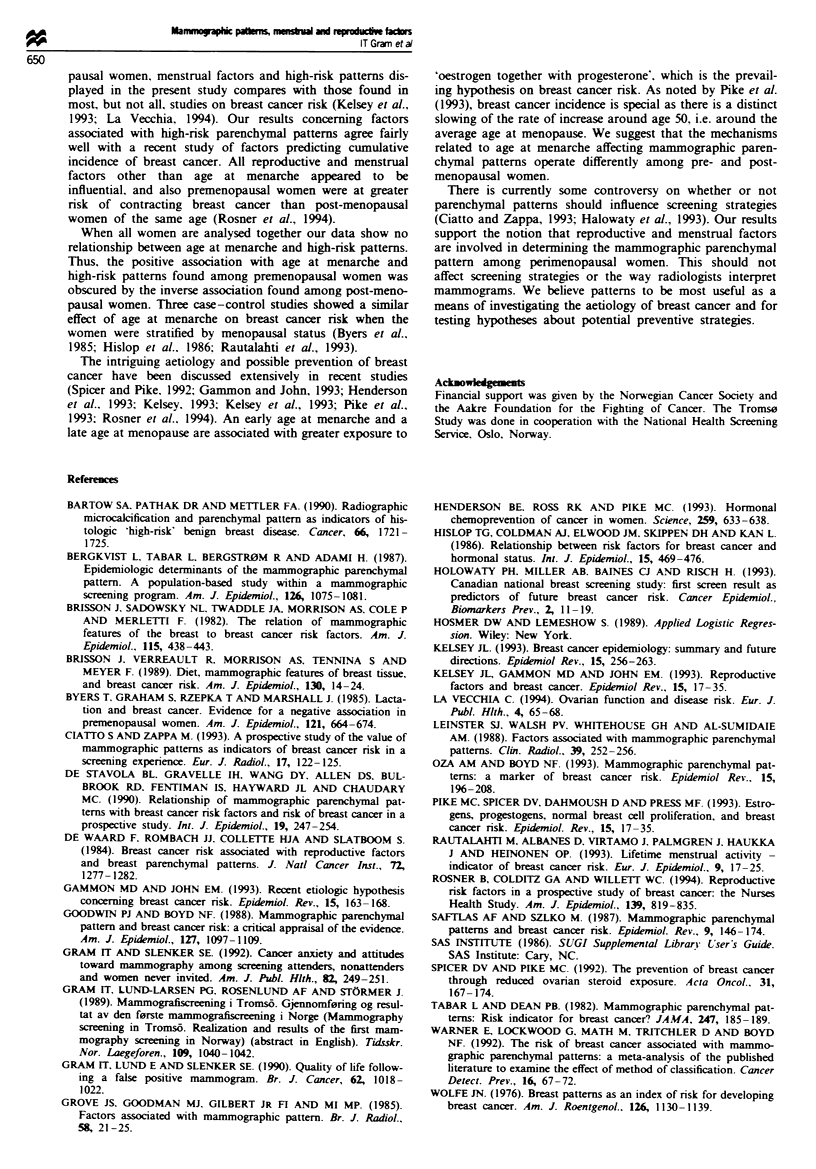# Reproductive and menstrual factors in relation to mammographic parenchymal patterns among perimenopausal women.

**DOI:** 10.1038/bjc.1995.128

**Published:** 1995-03

**Authors:** I. T. Gram, E. Funkhouser, L. Tabar

**Affiliations:** Institute of Community Medicine, University of Tromsö, Breivika, Norway.

## Abstract

The relationship between mammographic patterns and reproductive and menstrual factors was examined in 3640 Norwegian women, aged 40-56 years, participating in the Third Tromsö study conducted in 1986-87. Epidemiological data were obtained from questionnaires. The mammograms were categorised into five groups. This categorisation is based on anatomic-mammographic correlations, following three-dimensional (thick slice technique) histopathologic-mammographic comparisons, rather than simple pattern reading. Patterns 1-3 were combined into a low-risk group and patterns 4 and 5 into a high-risk group for analysis. Women who had more than four children were 90% less likely to have a high-risk pattern than nulliparous women (OR = 0.09, 95% CI 0.04-0.16) controlling for age, weight, height and menopausal status. Furthermore, those who first gave birth over 34 years of age were more than twice as likely to have a high-risk pattern than those giving birth in their teens (OR = 2.37, 95% CI 1.23-4.56) adjusting for parity. Among post-menopausal women, age at menarche was negatively (P for trend = 0.015) and late age at menopause positively (P for trend = 0.072) related to high-risk patterns. Among premenopausal women, age at menarche was positively related to high-risk patterns (P for trend = 0.001). Also, menopausal status rather than age was associated with high-risk patterns. These findings support the opinion that reproductive and menstrual factors are involved in determining the mammographic parenchymal pattern among perimenopausal women.


© 1995 Stockton Press All rights reserved 0007–0920/95 $9.00

### Reproductive and menstrual factors in relation to mammographic parenchymal patterns among perimenopausal women

IT Gram[1], E Funkhouser[2] and L Tabar[3]

[1]*Institute of Community Medicine, University of Tromsö, Breivika N-9037 Norway;* [2]*Department of Epidemiology, School of Public Health, University of Alabama at Birmingham, Alabama 35294, USA;* [3]*Department of Mammography, Falun Central Hospital, S-791 82 Falun, Sweden.*

**Summary** The relationship between mammographic patterns and reproductive and menstrual factors was examined in 3640 Norwegian women, aged 40–56 years, participating in the Third Tromsö study conducted in 1986–87. Epidemiological data were obtained from questionnaires. The mammograms were categorised into five groups. This categorisation is based on anatomic–mammographic correlations, following three-dimensional (thick slice technique) histopathologic–mammographic comparisons, rather than simple pattern reading. Patterns 1–3 were combined into a low-risk group and patterns 4 and 5 into a high-risk group for analysis. Women who had more than four children were 90% less likely to have a high-risk pattern than nulliparous women (OR = 0.09, 95% CI 0.04–0.16) controlling for age, weight, height and menopausal status. Furthermore, those who first gave birth over 34 years of age were more than twice as likely to have a high-risk pattern than those giving birth in their teens (OR = 2.37, 95% CI 1.23–4.56) adjusting for parity. Among post-menopausal women, age at menarche was negatively (*P* for trend = 0.015) and late age at menopause positively (*P* for trend = 0.072) related to high-risk patterns. Among premenopausal women, age at menarche was positively related to high-risk patterns (*P* for trend = 0.001). Also, menopausal status rather than age was associated with high-risk patterns. These findings support the opinion that reproductive and menstrual factors are involved in determining the mammographic parenchymal pattern among perimenopausal women.

**Keywords:** mammography; Norway; parenchymal breast patterns; parity; reproductive factors; menarche; menopause; risk factors

There is now a large amount of evidence showing that mammographic parenchymal patterns are a marker of breast cancer risk (Saftlas and Szlko, 1987; Goodwin and Boyd, 1988; Brisson *et al.*, 1989; Warner *et al.*, 1992; Holowaty *et al.*, 1993; Oza and Boyd, 1993).

The relationship between these mammographic features and risk factors for breast cancer has also been investigated. A consistent inverse association between high-risk parenchymal pattern and age, weight and parity has been reported. However, data regarding an association between these patterns and age at menarche, age at menopause and age at first birth are inconclusive (Bergkvist *et al.*, 1987; Saftlas and Szlko, 1987; De Stavola *et al.*, 1990; Oza and Boyd, 1993).

The aetiological mechanisms by which mammographic parenchymal pattern is associated with the occurrence of breast cancer are unknown. The purpose of this study was to examine whether reproductive and menstrual factors are involved in determining the mammographic parenchymal pattern among perimenopausal women in a population-based study.

### Materials and methods

Mammographic screening was a part of the Third Tromsö study, a health survey of men and women living in Tromsö, Norway, in 1986 and 1987. Women born between 1930 and 1966 were invited to participate. Those aged 40 or older ($n = 4323$) were offered free mammography, with 3653 (85%) accepting. Details of the screening and case finding procedures are given elsewhere (Gram *et al.*, 1989, 1990).

The participants in the Third Tromsö survey completed one questionnaire at the screening facility and another at home. The first questionnaire concerned disease history and aspects of living habits. The screenees also had their height and weight measured. The second questionnaire elicited information on reproductive variables, dietary habits, previous diseases and social and psychological conditions. The participants were asked to return this questionnaire by mail.

All screening mammograms, excluding those which detected breast cancer, were classified according to a system developed by one of the authors (LT). No details other than the date of birth were known to him. The Tabar system, patterns 1–5, is a further development of the mammographic parenchymal pattern classification. This classification is based on anatomic–mammographic correlations following three-dimensional (thick slice technique) histopathologic– mammographic comparisons rather than simple pattern reading as in the Wolfe system (Wolfe, 1976). Four out of the five patterns in the Tabar system may directly be compared to the patterns in the Wolfe system, i.e. low-risk patterns 2 and 3 equate to N1 and P1 respectively, and high-risk patterns 4 and 5 are equivalent to P2 and DY respectively. Thus, the important difference between the Wolfe and the Tabar classification concerns Tabar's pattern 1, which is described in detail below.

Pattern 1 describes the three main characteristics of normal fibroglandular tissue: (1) evenly scattered 1–2 mm nodular densities, corresponding to normal terminal ductal lobular units; (2) Cooper's ligament and concave parenchymal contour; and (3) oval-shaped/circular lucent areas, corresponding to fatty replacement, this resulting in a harmonious structure. Pattern 1 may evolve to pattern 2 following fatty replacement, or pattern 3, i.e. a retroareolar linear pattern, as a result of periductal elastosis or fatty involution. Wolfe completed his patterns description with a fifth pattern, QDY, which may be compared to Tabar pattern 1 (Wolfe, 1976). Wolfe states that the QDY pattern should be used for women below age 45 whose degree of dysplasia is not severe and that these patterns often regress with age to less dense patterns, Wolfe's P2 or P1 (Wolfe, 1976). We combined patterns 1–3 into a low-risk group and patterns 4 and 5 into a high-risk group for analysis (Tabar and Dean, 1982; Bergkvist *et al.*, 1987).

Prevalence odds ratios (ORs) were used to express the degree of association between mammographic patterns and

Correspondence: IT Gram
Received 23 February 1994; revised 6 July 1994; accepted 24 October 1994

breast cancer risk factors. Each of the following factors was evaluated as a potential confounder of the risk factor/high-risk pattern relation: age, weight and height. The odds ratios for each of these factors were estimated in both univariate and multivariate analyses for all women and also subdivided by menopausal status. A logistic regression model was used to allow for the effects of several potential confounders. Statistical trend tests were obtained by creating an ordinal exposure variable with equally spaced scores and including it in the logistic regression model (Hosmer and Lemeshow, 1989).

Results were considered as statistically significant if the $P$-value was 0.05 or less, and 95% confidence intervals (CIs) are reported throughout the paper. The multiple logistic regression analyses were performed using the Proc Logist procedure in the SAS statistical package (SAS Institute, 1986).

### Results

Table I displays selected attributes of the women at the time of the screening according to menopausal status. Premenopausal women were on average younger and were slightly younger at menarche and at first birth than post-menopausal women. More premenopausal women were parous, but they had, on average, fewer children than did post-menopausal women (Table I).

Patterns were classified for 3640 women as follows: 1659 as pattern 1, 857 as pattern 2, 401 as pattern 3, 354 as pattern 4 and 369 as pattern 5. Thus, 20% (723) of the women were classified as having a high-risk pattern. Table II displays the prevalence and crude odds ratio estimates of high-risk patterns by age and menopausal status. Premenopausal women were more likely than post-menopausal women to have a high-risk pattern (OR = 1.3, 95% CI 1.1–1.5). This association was present when stratified by age and was strongest in the youngest age group. Though younger women were more likely to have a high-risk pattern than older women, the association of age with pattern was weak or not present when stratified by menopausal status (Table II).

Weight was inversely and height positively associated with high-risk patterns and are together with age adjusted for in all analyses. A multivariate model that included terms for age, weight, height (all three continuous), menopausal status (premenopausal, post-menopausal), parity (0, 1–2, 3–4, 5 +) and age at first birth (<20, 20–24, 25–29, 30–34, 35 +) as independent variables and high-risk patterns as the dependent variable was fitted.

The odds ratio estimates of high-risk patterns according to reproductive and menstrual factors are shown in Tables III and IV respectively.

Table III shows that parous women were less likely to have high-risk patterns than nulliparous women (OR = 0.36, 95% CI 0.29–0.47) (Table III). Women having more than four children were 90% less likely to have a high-risk pattern than nulliparous women (OR = 0.09, 95% CI 0.04–0.16). Dose-response was evaluated among parous women. An ordinal trend test across the three levels of parity (1–2, 3–4, 5 +), displayed in Table III, yields a $P$-value of less than 0.001.

Table III shows that the ORs pertaining to age at first birth were highest among women who had their first child at an older age, controlling for parity. Nulliparous women were added as the last category in the same model, after age group 35 +. The results of this analysis showed that nulliparous women were more than twice as likely to have a high-risk pattern compared with women who had their first child in their teens (OR = 2.37, 95% CI 1.02–5.50). A statistical trend test for age at first birth (with five categories among parous women) adjusting for parity yielded a $P$-value less than 0.001. Adding the nulliparous women as the sixth category gave a similar result.

The trend with age at first birth was evident among both women with few (1 or 2) children ($P = 0.0001$) and women with many (3 +) children ($P = 0.006$). The trend with parity (three levels: 1–2, 3–4, 5 +) was also revealed among women with young (<24 years) age at first birth ($P = 0.001$) and among those with late (30 + years) age at first birth ($P = 0.014$). Table IV displays the relationship between age at menarche (four levels: <12, 12–13, 14–15, 16 +) and high-risk patterns stratified by menopausal status adjusted for age, height, weight and parity (Table IV). Among premenopausal women, those whose age at menarche was 16 + were more than twice as likely to have a high-risk pattern (OR = 2.4, 95% CI 1.3–4.5) than those whose age at menarche was less than 12 years. Among post-menopausal women, those whose age at menarche was 16 + were 80% less likely to have a high-risk pattern (OR = 0.2, 95% CI 0.1–0.6) than those with an early age at menarche. Dose-response was evaluated using the four levels of age at menarche displayed in Table IV. The trend between age at menarche and high-risk patterns was evident among both premenopausal ($P<0.001$) and post-menopausal women ($P = 0.015$) (Table IV).

**Table I** Selected attributes at screening by menopausal status given as mean (s.d.) and per cent, Tromsø, Norway

| | *Premenopausal* n = *2303* | *Post-menopausal* n = *1337* |
|---|---|---|
| Age (years) | 44.3 (3.6) | 51.8 (4.2) |
| Parous (%) | 92.0 | 87.1 |
| No. of children | 2.4 (1.3) | 2.6 (1.7) |
| Age at first birth[a] (years) | 23.1 (4.2) | 23.2 (3.9) |
| Age at menarche (years) | 13.3 (1.4) | 13.6 (1.4) |
| Age at menopause[b] (years) | | 46.5 (5.4) |

[a]Among parous women. [b]Among post-menopausal women.

**Table II** Prevalence (%) and crude odds ratio estimates of high-risk mammographic patterns[a] by age and menopausal status, Tromsø, Norway

| *Age* | *Population* n *(%)* | *Pre-menopausal* n *(%)* | *Post-menopausal* n *(%)* | *Odds ratio*[b] |
|---|---|---|---|---|
| 40–45 | 1428 (20.3) | 1331 (21.0) | 97 (11.3) | 2.1 (1.1–4.4)[c] |
| 46–50 | 1173 (20.1) | 824 (21.2) | 349 (17.5) | 1.3 (0.9–1.8) |
| 51–56 | 1039 (19.0) | 148 (25.0) | 891 (18.0) | 1.5 (1.0–2.3) |
| All | 3640 (20.0) | 2303 (21.3) | 1337 (17.4) | 1.3 (1.1–1.5) |

[a]High risk = patterns 4 and 5. [b]Post-menopausal women, reference category. [c]Numbers in parentheses are 95% confidence interval.

**Table III** Odds ratio estimates for high-risk mammographic patterns[a] according to reproductive factors surveyed at population screening, Tromsø, Norway

| *Reproductive factors* | *High*[a] *risk* | *Low*[b] *risk* | *Odds ratio*[c] | *Trend test* |
|---|---|---|---|---|
| Parous | | | | |
| No | 116 | 216 | 1.00 | |
| Yes | 544 | 2506 | 0.36 (0.29–0.47)[d] | |
| No. of children | | | | |
| 0 | 116 | 216 | 1.00 | $P<0.001$[e] |
| 1–2 | 322 | 1093 | 0.52 (0.40–0.68) | |
| 3–4 | 210 | 1160 | 0.29 (0.21–0.38) | |
| 5+ | 12 | 253 | 0.09 (0.04–0.16) | |
| Age at first birth[f] (years) | | | | |
| <20 | 60 | 475 | 1.00 | $P = 0.001$[g] |
| 20–24 | 293 | 1500 | 1.35 (0.98–1.84) | |
| 25–29 | 171 | 558 | 1.81 (1.27–2.58) | |
| 30–34 | 53 | 120 | 2.51 (1.53–4.12) | |
| 35+ | 22 | 50 | 2.37 (1.23–4.56) | |

[a]High risk, patterns 4 and 5. [b]Low risk, patterns 1, 2 and 3 (reference category). [c]Adjusted for age, menopausal status, weight and height. [d]Numbers in parentheses represent 95% confidence interval. [e]Trend test among parous women (three categories of parity). [f]Also adjusted for number of children. [g]Trend test among parous women (five categories of age at first parity).

**Table IV** Odds ratio estimates for high-risk mammographic patterns[a] according to menstrual factors surveyed at population screening, Tromsø, Norway

| *Menstrual factors* | *High[a] risk* | *Low[b] risk* | *Odds ratio[c]* | *Trend test* |
|---|---|---|---|---|
| Age at menarche[d] years | | | | |
| <12 | 29 | 155 | 1.00 | $P = 0.001$ |
| 12–13 | 178 | 792 | 1.1 (0.7–1.8)[e] | |
| 14–15 | 193 | 629 | 1.4 (0.9–2.1) | |
| 16+ | 38 | 67 | 2.4 (1.3–4.5) | |
| Age at menarche[f] (years) | | | | |
| <12 | 13 | 58 | 1.00 | $P = 0.001$ |
| 12–13 | 88 | 410 | 0.6 (0.3–1.2) | |
| 14–15 | 101 | 468 | 0.5 (0.3–1.1) | |
| 16+ | 12 | 82 | 0.2 (0.1–0.6) | |
| Age at menopause[f] (years) | | | | |
| <45 | 56 | 310 | 1.00 | $P = 0.072$ |
| 45–52 | 157 | 717 | 1.3 (0.9–2.0) | |
| 53+ | 19 | 78 | 1.5 (0.7–3.3) | |

[a]High risk, patterns 4 and 5. [b]Low risk, patterns 1, 2 and 3 (reference category). [c]Adjusted for age, weight, height and number of children. [d]Among premenopausal women. [e]Numbers in parentheses represent 95% confidence interval. [f]Among post-menopausal women.

To give more stable estimates of the association between age at menarche and high-risk patterns, age at menarche was divided in two categories: 13 years or less and more than 13 years. The directions of the associations were the same as indicated in Table IV. Among premenopausal women, those whose age at menarche was >13 were 35% more likely to have a high-risk pattern (OR = 1.35, 95% CI 1.08–1.69), and among post-menopausal women those whose age at menarche was >13 were 25% less likely to have a high-risk pattern (OR = 0.75, 95% CI 0.54–1.04) than those with a younger age at menarche.

Table IV shows that women with a later age at menopause were more likely to have high-risk patterns than those with an early menopause (Table IV). Stratified by age groups, women aged 45–52 years at menopause were 30% more likely (OR = 1.3, 95% CI 0.9–2.0) and those aged over 52 were 50% more likely (OR = 1.5, 95% CI 0.7–3.2) to have high-risk patterns than those having an early menopause (i.e. less than 45 years). A statistical trend test across the three levels described did not achieve statistical significance ($P = 0.072$).

### Discussion

This study confirms that parity is inversely associated with high-risk mammographic patterns. In addition, an independent positive association of age at first birth and high-risk patterns is demonstrated. Age at menarche is positively associated with high-risk patterns among premenopausal women, whereas the inverse association is displayed among post-menopausal women. Furthermore, our results indicate an association between late age at menopause and high-risk patterns previously not shown. The study also suggests that menopausal status rather than age appears to be most closely related to mammographic patterns among perimenopausal women.

A limitation of our study is that the temporal relationship bewteen the factors studied and the mammographic patterns is unknown. The use of recalled age at menarche, age at menopause and age at first birth may cause non-differential misclassification, and thereby attenuate the real association. We consider therefore our estimates to be conservative ones.

We attribute much of the consistency of the findings to the Tabar system in which one of the patterns previously classified as a high-risk is now classified as a low-risk pattern. The mammograms were classified by an experienced mammographer (LT) who had no knowledge of the risk factors surveyed, and we were also able to adjust for potential confounding variables. The study is population based, and the attenders did not differ from the non-attenders with respect to the risk factors studied (Gram and Slenker, 1992).

A causal interpretation of the association between reproductive factors and high-risk patterns is supported by the presence of a dose–response relation between the various levels of parity and age at first birth with high-risk patterns. These associations are present when stratified both by menopausal status and by each other. The effects are not confounded by weight or height as this has been adjusted for in the multivariate analyses.

Several studies have found an association with either parity or age at first birth and parenchymal patterns (Bergkvist *et al.*, 1987; Saftlas and Szlko, 1987; Leinster *et al.*, 1988; Brisson *et al.*, 1989; De Stavola *et al.*, 1990). However, only in the study by De Waard *et al.* (1984) is an independent effect of both aspects of reproductive life revealed, which remains after adjusting for Quetelet's index.

Our study shows an association between age at menarche and high-risk patterns stratified by menopausal status adjusting simultaneously for parity, age, weight and height One of the two studies listed in the review by Saftlas and Szlko (1987) also demonstrates a positive association between age at menarche and high-risk patterns. In a later analyses, expanded to include more than 5000 Guernsey women stratified by menopausal status, the positive association between age at menarche and high-risk patterns among premenopausal women was totally explained by adiposity. In post-menopausal women, the positive association remained statistically significant after adjusting for age, parity and Quetelet's index (De Stavola *et al.*, 1990). In another study of 5319 screenees, there was no correlation between high-risk patterns and age at menarche for either menopausal category when breast size, weight, late age at first pregnancy, prior biopsy and history of cyclical breast pain were included in the model (Leinster *et al.*, 1988). The positive association between age at menarche and high-risk patterns found among premenopausal women in our study and among post-menopausal Guernsey women may be due to chance.

Our study shows an association between age at menarche and high-risk patterns stratified by menopausal status adjusting simultaneously for parity, age, weight and height. One of the two studies listed in the review by Saftlas and Szlko (1987) also demonstrates a positive association between age at menarche and high-risk patterns. In a later analysis, narrow limits. We find no overall effect of age on high-risk patterns. However, at a given age, premenopausal women are at greater risk of high-risk patterns than post-menopausal women of the same age. Two other studies reported similar results (Grove *et al.*, 1985; Leinster *et al.*, 1988). In the study from Guernsey, the proportion of high-risk patterns was found to peak around menopause (De Stavola *et al.*, 1990). Among the studies that did not stratify by menopausal status, the Swedish investigators reported a peak of high-risk patterns in the age group 46–50 years, whereas the other researchers found decreasing proportions of high-risk patterns with increasing age (Bergkvist *et al.*, 1987; Saftlas and Szlko, 1987; Brisson *et al.*, 1989; Bartow *et al.*, 1990; Ciatto and Zappa, 1993).

Twenty per cent of the mammograms in our study were classified as high-risk patterns, while the corresponding figure in most studies using Wolfe's classification is between 30% and 70% (Saftlas and Szlko, 1987; Ciatto and Zappa, 1993; Holowaty *et al.*, 1993). All our high-risk patterns would be classified as such in the Wolfe system. However, one of our low-risk patterns (pattern 1) would most likely be classified as a high-risk pattern, i.e. either a P2 or a DY depending on the woman's age according to the Wolfe system (Wolfe, 1976). Thus, the low proportion of high-risk patterns in our study is due not to inter- and intra-observer variations, but to this difference in the classification system. This should be kept in mind when the results from our study are compared with those of others.

The relation between reproductive and, among post-menopausal women, menstrual factors and high-risk patterns displayed in the present study compares with those found in most, but not all, studies on breast cancer risk (Kelsey *et al.*, 1993; La Vecchia, 1994). Our results concerning factors associated with high-risk parenchymal patterns agree fairly well with a recent study of factors predicting cumulative incidence of breast cancer. All reproductive and menstrual factors other than age at menarche appeared to be influential, and also premenopausal women were at greater risk of contracting breast cancer than post-menopausal women of the same age (Rosner *et al.*, 1994).

When all women are analysed together our data show no relationship between age at menarche and high-risk patterns. Thus, the positive association with age at menarche and high-risk patterns found among premenopausal women was obscured by the inverse association found among post-menopausal women. Three case-control studies showed a similar effect of age at menarche on breast cancer risk when the women were stratified by menopausal status (Byers *et al.*, 1985; Hislop *et al.*, 1986; Rautalahti *et al.*, 1993).

The intriguing aetiology and possible prevention of breast cancer have been discussed extensively in recent studies (Spicer and Pike, 1992; Gammon and John, 1993; Henderson *et al.*, 1993; Kelsey, 1993; Kelsey *et al.*, 1993; Pike *et al.*, 1993; Rosner *et al.*, 1994). An early age at menarche and a late age at menopause are associated with greater exposure to 'oestrogen together with progesterone', which is the prevailing hypothesis on breast cancer risk. As noted by Pike *et al.* (1993), breast cancer incidence is special as there is a distinct slowing of the rate of increase around age 50, i.e. around the average age at menopause. We suggest that the mechanisms related to age at menarche affecting mammographic parenchymal patterns operate differently among pre- and post-menopausal women.

There is currently some controversy on whether or not parenchymal patterns should influence screening strategies (Ciatto and Zappa, 1993; Haloway *et al.*, 1993). Our results support the notion that reproductive and menstrual factors are involved in determining the mammographic parenchymal pattern among perimenopausal women. This should not affect screening strategies or the way radiologists interpret mammograms. We believe patterns to be most useful as a means of investigating the aetiology of breast cancer and for testing hypotheses about potential preventive strategies.

**Acknowledgements**
Financial support was given by the Norwegian Cancer Society and the Aakre Foundation for the Fighting of Cancer. The Tromsø Study was done in cooperation with the National Health Screening Service, Oslo, Norway.

**References**

BARTOW SA, PATHAK DR AND METTLER FA. (1990). Radiographic microcalcification and parenchymal pattern as indicators of histologic 'high-risk' benign breast disease. *Cancer*, **66**, 1721-1725.

BERGKVIST L, TABAR L, BERGSTRØM R AND ADAMI H. (1987). Epidemiologic determinants of the mammographic parenchymal pattern. A population-based study within a mammographic screening program. *Am. J. Epidemiol.*, **126**, 1075-1081.

BRISSON J, SADOWSKY NL, TWADDLE JA, MORRISON AS, COLE P AND MERLETTI F. (1982). The relation of mammographic features of the breast to breast cancer risk factors. *Am. J. Epidemiol.*, **115**, 438-443.

BRISSON J, VERREAULT R, MORRISON AS, TENNINA S AND MEYER F. (1989). Diet, mammographic features of breast tissue, and breast cancer risk. *Am. J. Epidemiol.*, **130**, 14-24.

BYERS T, GRAHAM S, RZEPKA T AND MARSHALL J. (1985). Lactation and breast cancer. Evidence for a negative association in premenopausal women. *Am. J. Epidemiol.*, **121**, 664-674.

CIATTO S AND ZAPPA M. (1993). A prospective study of the value of mammographic patterns as indicators of breast cancer risk in a screening experience. *Eur. J. Radiol.*, **17**, 122-125.

DE STAVOLA BL, GRAVELLE IH, WANG DY, ALLEN DS, BULBROOK RD, FENTIMAN IS, HAYWARD JL AND CHAUDARY MC. (1990). Relationship of mammographic parenchymal patterns with breast cancer risk factors and risk of breast cancer in a prospective study. *Int. J. Epidemiol.*, **19**, 247-254.

DE WAARD F, ROMBACH JJ, COLLETTE HJA AND SLATBOOM S. (1984). Breast cancer risk associated with reproductive factors and breast parenchymal patterns. *J. Natl Cancer Inst.*, **72**, 1277-1282.

GAMMON MD AND JOHN EM. (1993). Recent etiologic hypothesis concerning breast cancer risk. *Epidemiol. Rev.*, **15**, 163-168.

GOODWIN PJ AND BOYD NF. (1988). Mammographic parenchymal pattern and breast cancer risk: a critical appraisal of the evidence. *Am. J. Epidemiol.*, **127**, 1097-1109.

GRAM IT AND SLENKER SE. (1992). Cancer anxiety and attitudes toward mammography among screening attenders, nonattenders and women never invited. *Am. J. Publ. Hlth.*, **82**, 249-251.

GRAM IT, LUND-LARSEN PG, ROSENLUND AF AND STÖRMER J. (1989). Mammografiscreening i Tromsö. Gjennomføring og resultat av den første mammografiscreening i Norge (Mammography screening in Tromsö. Realization and results of the first mammography screening in Norway) (abstract in English). *Tidsskr. Nor. Laegeforen.*, **109**, 1040-1042.

GRAM IT, LUND E AND SLENKER SE. (1990). Quality of life following a false positive mammogram. *Br. J. Cancer*, **62**, 1018-1022.

GROVE JS, GOODMAN MJ, GILBERT JR FI AND MI MP. (1985). Factors associated with mammographic pattern. *Br. J. Radiol.*, **58**, 21-25.

HENDERSON BE, ROSS RK AND PIKE MC. (1993). Hormonal chemoprevention of cancer in women. *Science*, **259**, 633-638.

HISLOP TG, COLDMAN AJ, ELWOOD JM, SKIPPEN DH AND KAN L. (1986). Relationship between risk factors for breast cancer and hormonal status. *Int. J. Epidemiol.*, **15**, 469-476.

HOLOWATY PH, MILLER AB, BAINES CJ AND RISCH H. (1993). Canadian national breast screening study: first screen result as predictors of future breast cancer risk. *Cancer Epidemiol., Biomarkers Prev.*, **2**, 11-19.

HOSMER DW AND LEMESHOW S. (1989). *Applied Logistic Regression*. Wiley: New York.

KELSEY JL. (1993). Breast cancer epidemiology: summary and future directions. *Epidemiol Rev.*, **15**, 256-263.

KELSEY JL, GAMMON MD AND JOHN EM. (1993). Reproductive factors and breast cancer. *Epidemiol Rev.*, **15**, 17-35.

LA VECCHIA C. (1994). Ovarian function and disease risk. *Eur. J. Publ. Hlth.*, **4**, 65-68.

LEINSTER SJ, WALSH PV, WHITEHOUSE GH AND AL-SUMIDAIE AM. (1988). Factors associated with mammographic parenchymal patterns. *Clin. Radiol.*, **39**, 252-256.

OZA AM AND BOYD NF. (1993). Mammographic parenchymal patterns: a marker of breast cancer risk. *Epidemiol Rev.*, **15**, 196-208.

PIKE MC, SPICER DV, DAHMOUSH D AND PRESS MF. (1993). Estrogens, progestogens, normal breast cell proliferation, and breast cancer risk. *Epidemiol. Rev.*, **15**, 17-35.

RAUTALAHTI M, ALBANES D, VIRTAMO J, PALMGREN J, HAUKKA J AND HEINONEN OP. (1993). Lifetime menstrual activity – indicator of breast cancer risk. *Eur. J. Epidemiol.*, **9**, 17-25.

ROSNER B, COLDITZ GA AND WILLETT WC. (1994). Reproductive risk factors in a prospective study of breast cancer: the Nurses Health Study. *Am. J. Epidemiol.*, **139**, 819-835.

SAFTLAS AF AND SZLKO M. (1987). Mammographic parenchymal patterns and breast cancer risk. *Epidemiol. Rev.*, **9**, 146-174.

SAS INSTITUTE (1986). *SUGI Supplemental Library User's Guide*. SAS Institute: Cary, NC.

SPICER DV AND PIKE MC. (1992). The prevention of breast cancer through reduced ovarian steroid exposure. *Acta Oncol.*, **31**, 167-174.

TABAR L AND DEAN PB. (1982). Mammographic parenchymal patterns: Risk indicator for breast cancer? *JAMA*, **247**, 185-189.

WARNER E, LOCKWOOD G, MATH M, TRITCHLER D AND BOYD NF. (1992). The risk of breast cancer associated with mammographic parenchymal patterns: a meta-analysis of the published literature to examine the effect of method of classification. *Cancer Detect. Prev.*, **16**, 67-72.

WOLFE JN. (1976). Breast patterns as an index of risk for developing breast cancer. *Am. J. Roentgenol.*, **126**, 1130-1139.